# The Interaction Effect of Age, Initial Rhythm, and Location on Outcomes After Out-of-Hospital Cardiac Arrest: A Retrospective Cohort Study

**DOI:** 10.3390/jcm13216426

**Published:** 2024-10-26

**Authors:** Łukasz Lewandowski, Aleksander Mickiewicz, Kamil Kędzierski, Paweł Wróblewski, Mariusz Koral, Grzegorz Kubielas, Jacek Smereka, Michał Czapla

**Affiliations:** 1 Department of Medical Biochemistry, Wroclaw Medical University, 50-367 Wrocław, Poland; lukasz.lewandowksi@umw.edu.pl; 2 Department of Emergency Medical Service, Faculty of Nursing and Midwifery, Wroclaw Medical University, 50-367 Wroclaw, Poland; aleksander.mickiewicz@umw.edu.pl (A.M.); kamil.kedzierski@umw.edu.pl (K.K.); pawel.wroblewski@umw.edu.pl (P.W.); jacek.smereka@umw.edu.pl (J.S.); 3 Medical Simulation Center, Faculty of Medicine, Wroclaw Medical University, 50-367 Wroclaw, Poland; mariusz.koral@umw.edu.pl; 4 Division of Healthcare Organization, Department of Nursing, Faculty of Nursing and Midwifery, Wroclaw Medical University, 50-367 Wroclaw, Poland; grzegorz.kubielas@umw.edu.pl; 5 Department of Health Care Services, Polish National Health Fund, Central Office in Warsaw, 02-528 Warsaw, Poland; 6 Group of Research in Care (GRUPAC), Faculty of Health Science, University of La Rioja, 26006 Logrono, Spain; 7 Institute of Heart Diseases, University Hospital, 50-566 Wroclaw, Poland

**Keywords:** out-of-hospital cardiac arrest, return of spontaneous circulation, age, sex, location, public places, initial rhythm

## Abstract

**Background**: Out-of-hospital cardiac arrest (OHCA) is a critical global health challenge and a leading cause of mortality. This study investigates the combined effect of initial cardiac arrest rhythm, patient age, and location on the return of spontaneous circulation (ROSC) in OHCA patients. **Methods**: This retrospective study analyzed medical records from the National Emergency Medical Service (EMS) in Poland between January 2021 and June 2022. Data from 33,636 patients with OHCA who received cardiopulmonary resuscitation (CPR) at the scene were included. **Results**: Public incidents were associated with higher ROSC rates (54.10% vs. 31.53%, *p* < 0.001). Initial shockable rhythms (VF/pVT) significantly increased the odds of ROSC (OR = 3.74, 95% CI 3.39–4.13, *p* < 0.001). Obesity decreased the odds of ROSC in at-home cases (OR = 0.85, 95% CI 0.73–0.99, *p* = 0.036) but had no significant effect in public cases. The effect of age on ROSC outcomes varied significantly depending on the location. In patients younger than 60 years, better ROSC outcomes were observed in at-home cases, while for those older than 60 years, the odds of ROSC were higher in public locations. Each additional year of age decreased the odds of ROSC by 1.62% in at-home incidents (*p* < 0.001) and by 0.40% in public incidents (*p* = 0.009). Sex differences were significant in public locations, with women having higher odds of ROSC compared to men (OR = 0.57, 95% CI 0.37–0.87, *p* = 0.009 for VF/pVT). **Conclusions**: The interaction between the location of OHCA, initial cardiac rhythm, and patient age significantly impacts ROSC outcomes. Public locations show higher ROSC rates, especially in cases with shockable rhythms (VF/pVT). Age modifies ROSC outcomes, with younger patients benefiting more at home, and older patients showing better outcomes in public places.

## 1. Introduction

Out-of-hospital cardiac arrest (OHCA) remains a major global health challenge and a leading cause of mortality, despite significant advancements in medicine [1]. The European Registry of Cardiac Arrest (EuReCa) data reveal that the incidence of OHCA in Europe ranges from 67 to 170 cases per 100,000 individuals annually [2]. In Poland, the occurrence varies regionally from 58 to 84 cases per 100,000 residents [3].

Prompt response is crucial in OHCA management. Before the arrival of emergency medical services (EMS), immediate initiation of cardiopulmonary resuscitation (CPR by bystanders and the availability of automated external defibrillators (AEDs) are critical [4]. Unfortunately, according to a report by the American Heart Association (AHA), only about 40% of patients with OHCA receive CPR from witnesses before the arrival of the EMS [5]. The frequency of bystander CPR in European countries is markedly inconsistent, averaging 58% with a range from 13% to 83% [2].

A meta-analysis by Yan et al. highlighted that spontaneous return of circulation (ROSC) occurs on-site in less than 30% of OHCA cases. Approximately 22% of patients survive the transport to the hospital, yet survival rates drop to below 9% at hospital discharge, and only 7.7% survive up to one year [6]. The analysis further demonstrated a higher survival rate to hospital discharge in cases where CPR was initiated by a bystander.

Notably, OHCAs occurring in public places have been associated with a higher probability of ROSC [7,8,9,10]. This increased likelihood is attributed to the rapid initiation of CPR, quicker EMS response, and public access to AEDs [11,12,13,14]. Particularly, survival rates are higher in densely populated public areas [15]. Our research in Poland corroborates these findings, showing nearly a 50% increase in ROSC odds when OHCA occurs in public places [16]. However, arrests in residential homes and high-rise settings, even when witnessed, have been consistently associated with worse outcomes [17].

The aim of this study is to investigate the combined effect of the cardiac arrest (CA) initial rhythm, patient age, and location of occurrence (public vs. non-public places) on ROSC in OHCA patients. The rationale behind this approach lies in the hypothesis that these factors may interact in ways that significantly influence ROSC outcomes. By analyzing these variables together, we hope to provide a more comprehensive understanding of how demographic and situational factors affect the likelihood of ROSC and, ultimately, patient survival.

## 2. Materials and Methods

### 2.1. Study Design and Setting

This study is a retrospective analysis of medical records from patients treated by EMS in Poland between January 2021 and June 2022. The data were obtained from the Command Support System of the National Emergency Medical Service and made available by the Emergency Medical Services Monitoring Centre with permission from the Polish Ministry of Health. The data were based on the documentation of EMS interventions, which is electronically entered by EMS personnel using tablet devices with specialized software immediately after completing medical procedures. The data are stored and managed in the Emergency Medical Services Monitoring Centre.

### 2.2. Study Population

**Figure 1:**
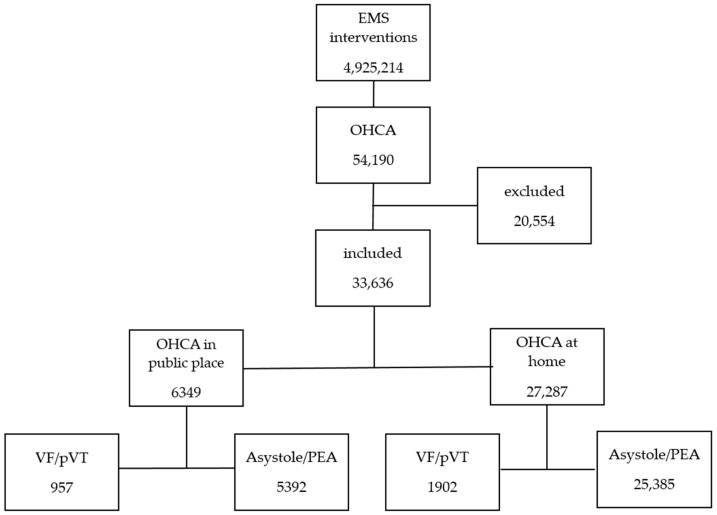
Flow chart of the study. Abbreviations: OHCA, out-of-hospital-cardiac-arrest; VF: ventricular fibrillation; pVT: pulseless ventricular tachycardia; PEA: pulseless electrical activity.

From the available database of 4,925,214 EMS calls, 54,190 patients with a diagnosis of cardiac arrest (ICD I10-I46) who received CPR at the scene were selected. Patients who had suffered OHCA as a result of suicide attempt, crime, trauma, etc., who had died before the arrival of EMS, and who had no information in their medical records about the initial rhythm of OHCA were then excluded from the study. Finally, data from 33,636 patients were analyzed (Figure 1). Patients were divided based on the occurrence of OHCA in public and non-public places. In this study, “public places” were defined as non-residential locations, including open public areas such as parks, streets, and public transportation stations, as well as workplaces, commercial areas, and other non-residential settings. “At-home” or “non-public places” referred exclusively to private residences. Data such as sex, age, comorbidities (hypertension (HT), diabetes mellitus (DM), heart failure (HF), obesity), and history of acute coronary syndrome (ACS) and stroke were analyzed.

### 2.3. Data Analysis

Data preprocessing and visualization was performed with Python 3.10.6 (packages: numpy 1.23.5, pandas 2.0.1, matplotlib 3.7.1, seaborn 0.12.2, forestplot 0.3.1) in Visual Studio Code 1.80.0. STATISTICA 13.3 on the license of Wroclaw Medical University was used for statistical modelling.

The α value used for statistical inference was 0.05. Classic comparisons were performed with the *t*-test (with Cochran–Cox correction if there was a violation of homoscedasticity assumption, as assessed with the Levene test) or the χ^2^ test, depending on the type of compared data (continuous or categorical). Logistic regression (binomial distribution of the predicted variable, link function: logit, dummy coding) was used for statistical modelling, to analyze the odds of ROSC. Pre-assumption of linearity vs. log(odds) was checked with the Box–Tidwell test. Full report concerning the analyzed logistic regression models is given in Supplementary Materials (Tables S1 and S2). Goodness of fit was assessed based on deviance, Akaike Information Criterion, and Bayesian Information Criterion. Multicollinearity was assessed with use of variance inflation factor (VIF) computed with Python (variance_inflation_factor from statsmodels.stats.outliers_influence). The VIFs of each variable are featured in Supplementary Materials (Table S3). Records with incomplete or missing data were excluded from analysis.

The three-way interaction featured in the last subsection featuring the results was contextually chosen based on its significance in the context of the type 3 likelihood ratio (LR) test performed on the full factorial model (up to three-way interactions). The selected interactions were then explored with the use of full factorial models containing the features from the analyzed interactions. The full set of conditional odds ratios was estimated based on the variance–covariance matrix of the full factorial models.

## 3. Results

### 3.1. Subsection Feature-Wise Contrasts in the Context of the Variable Emergency Location and Type of Initial Rhythm

Stratifying the population based solely on location (Table 1) revealed that younger individuals were more frequently subject to CA in a public place (*p* < 0.001). However, the distribution of individuals with different initial rhythms possibly influenced this observation as VF/pVT, which was associated with younger age (Table 2), was over two-fold more frequent in public places (15.07% vs. 6.97%, Table 1). Incidence of CA in a public place was also associated with lower frequency of diabetes (14.76% vs. 16.16%, Table 1) and higher frequencies of heart infarction (4.79% vs. 3.80%, Table 1) and ROSC (54.10% vs. 31.53%, Table 1).

Stratification by both CA location and initial rhythm (Table 2) showed many similarities between the two CA locations (domicile and public) in comorbidity frequencies between asystole/PEA and VF/pVT groups. Regardless of the location, men were more prone to develop a VF/pVT initial rhythm (76.66% and 77.85% at home and in public places, respectively). Moreover, in both incidence locations, diabetes, hypertension, and heart failure were more frequently coexistent with the asystole/PEA rhythm, and heart infarction was markedly more frequently associated with the VF/pVT rhythm (Table 2).

There were two contrasts that were observed regarding different locations and initial rhythms. Firstly, individuals who developed CA at home were more likely to be obese (3.29% vs. 2.31%, Table 2). This was not observed in the group that developed CA in a public place (*p* ≈ 0.060, Table 2). Secondly, although it could be stated that VF/pVT was more likely to develop ROSC regardless of the CA location, the public incident location was associated with a higher frequency of ROSC among the individuals, regardless of the type of the initial rhythm (79.31% and 49.63% vs. 61.41% and 29.30% among the VF/pVT and asystole/PEA, public place vs. at home, respectively; Table 2).

### 3.2. Analysis of the Odds for ROSC in the Context of Incidence Location and Initial Rhythm—Insights from Multi-Factor-Adjusted Logistic Regression Models (No Interactions Analyzed)

Upon division of the population solely based on incidence location (Table 3, Table S1), one would observe that the fact that regardless of the location, the VF/pVT initial rhythm markedly (over 3.7-fold) increases the odds of ROSC compared to the asystole/PEA initial rhythm. Among the at-home cases, obesity decreased the odds of ROSC by 18.06% (*p* ≈ 0.036), while it did not modulate these odds among public cases (*p* ≈ 0.451). Moreover, exclusively among the individuals who developed CA in a public place, either stroke or heart infarction increased the odds of ROSC (by 28.2% and 44.9%, respectively). Moreover, the odds of ROSC were differently modulated by age depending on the CA location—each subsequent increase in age by one year decreased the odds by 1.62% in at-home cases (*p* < 0.001), or by 0.40% upon public incidence (*p* ≈ 0.009).

More complex stratification (Table 4 and Table S2) reveals that initial rhythm brought differences in how the aforementioned factors modulated the odds of ROSC. Among the at-home cases, groups of two different initial rhythms were comparable in age-associated ORs of ROSC (0.984 vs. 0.980 in asystole/PEA, VF/pVT, respectively). Among the public cases, age modulated the odds only among individuals of asystole/PEA rhythm (OR = 0.995, *p* ≈ 0.010). In individuals of VF/pVT rhythm and at home CA location, diabetes increased the ROSC odds by 39.9% (*p* ≈ 0.040). Exclusively in VF/pVT public cases of CA, men were of 74.21% lower odds compared to women (*p* ≈ 0.011). Moreover, exclusively among the asystole/PEA public cases of CA, heart infarction increased the odds by 43.0% (*p* ≈ 0.022).

### 3.3. Exploring the Three-Way Modulation of the Odds of ROSC by Sex, Location, and Initial Rhythm—Insights from a Multivariate, Full Factorial Model (Interactions up to Third Grade Analyzed)

**Figure 2:**
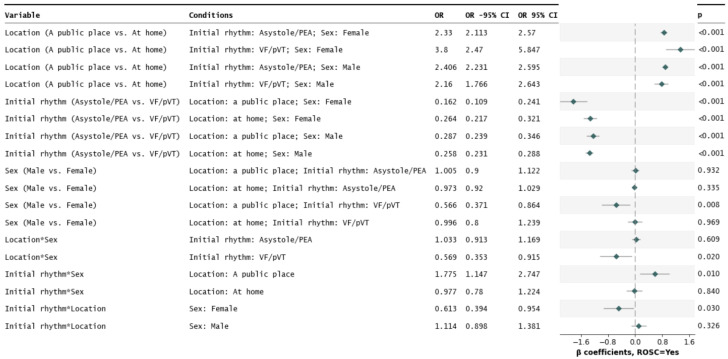
A three-way interaction between emergency location, sex, and cardiac arrest (CA) mechanism affecting the odds of ROSC. Based on a full factorial model with up to third-grade interactions (Table S4). This figure features odds ratios (ORs). Estimated odds of ROSC are visualized in Figure 3.

**Figure 3:**
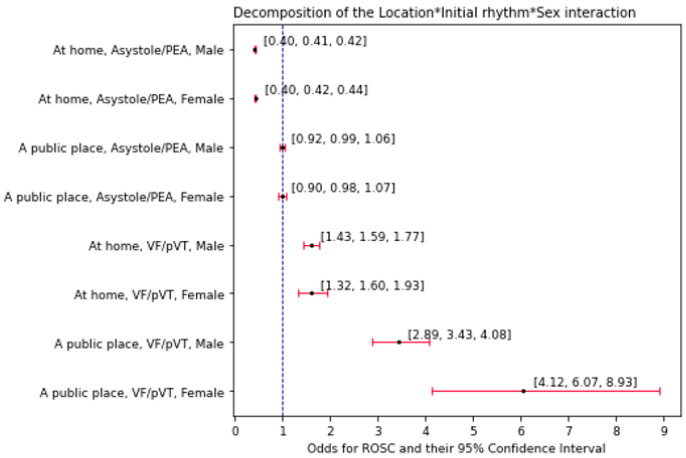
Modulation of odds of ROSC by sex, location, and initial rhythm. Estimated (based on the β_0_ intercept) from a full factorial model with up to third-grade interactions (Table S4). The ratios of these odds (ORs) are shown in Figure 2.

A full logistic regression model with effects and interactions reveals a three-way, multivariate simultaneous modulation of odds for ROSC by sex, incidence location, and initial rhythm (location × initial rhythm × sex, *p* ≈ 0.017, Table S4). The model was decomposed into lower-dimensional effects, which are shown in Figure 2 and Figure 3. Odds ratios and their changes upon different conditions are shown in Figure 2. Estimated odds (components of the aforementioned ORs) and their conditional change are shown in Figure 3. The interaction has been analyzed in three possible ways, as featured in the next subsections.

#### 3.3.1. Emergency Location as an Analyzed Effect in Variable Conditions Driven by CA Mechanism and Sex

Regardless of the CA mechanism and the occurrence of stroke, the public location of the emergency markedly (over two-fold) favored the incidence of ROSC compared to the domicile location. From the four possible combinations of CA mechanism and stroke categories, the highest OR between the two emergency locations was observed among the female VF/pVT individuals (OR = 3.80, *p* < 0.001). This value was approximately 1.63-fold higher than the respective OR estimated for men with the VF/pVT CA mechanism (*p* ≈ 0.030). In the case of the individuals of the asystole/PEA mechanism, sex did not alter these location-wise ORs (*p* ≈ 0.326).

#### 3.3.2. CA Mechanism as an Analyzed Effect in Variable Conditions Driven by the Emergency Location and Sex

Regardless of emergency location and the occurrence of stroke, the VF/pVT CA mechanism was always markedly (over three-fold) promoting ROSC compared to the asystole/PEA mechanism. This disproportion in odds was visibly greater among female individuals with a CA emergency in a public place (OR = 6.17, VF/pVT vs. asystole/PEA, *p* < 0.0001). If an emergency occurred in a public place, females showed approximately 1.77-fold higher OR (VF/pVT vs. asystole/PEA) compared to men (*p* ≈ 0.010). According to the model, sex would not alter these odds if the emergency was taking place at home (*p* ≈ 0.840).

#### 3.3.3. Sex as an Analyzed Effect in Variable Conditions Driven by the Emergency Location and CA Mechanism

The male vs. female difference in odds for ROSC was significant only in the case of individuals of the VF/pVT CA mechanism undergoing an emergency in a public place (male vs. female OR = 0.566, *p* ≈ 0.008). This occurrence resulted in significant two-way differences (interactions) based on the CA mechanism or emergency location.

### 3.4. How Age and Location, Together, Modulate the Odds of ROSC—Insights into Their 3rd-Degree Interaction with an Initial Rhythm

**Figure 4:**
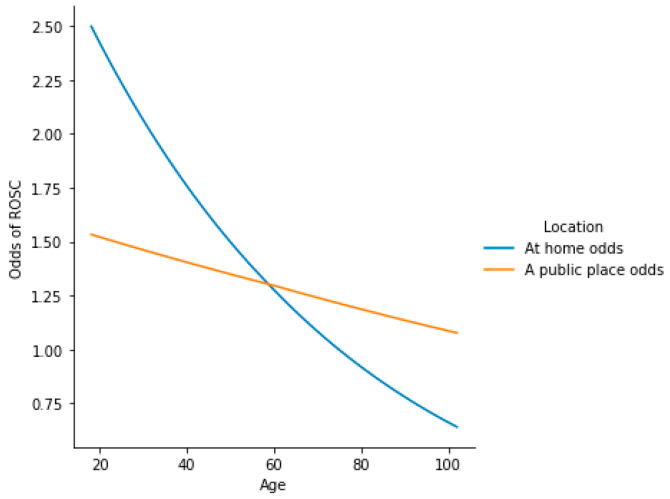
Location-wise differences in how age modulates the odds of ROSC among the asystole/PEA individuals, based on multivariate logistic regression models shown in Table S5. The change pattern in VF/pVT is identical, although all of the odds values would be 3.746-fold higher than those shown in this plot.

The above-mentioned difference in how age affected the odds of ROSC depending on location was analyzed with the use of a model featuring interactions (Table S5). Based on this, it could be stated that the initial rhythm does not significantly affect the way both location and age modulate the odds of ROSC (*p* ≈ 0.372). Thus, the baseline odds of ROSC, although differing by 3.746-fold (VF/pVT vs. asystole/PEA), would change in the same pattern, depending on location and age. This pattern, depicted for the asystole/PEA rhythm, is shown in Figure 4.

**Figure 5:**
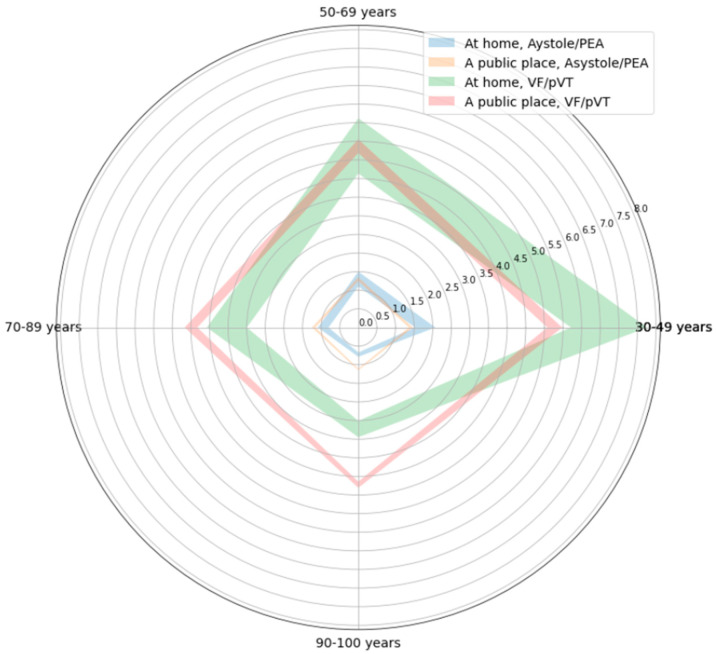
Ranges of odds of ROSC for different age groups, depending on initial rhythm and location of the incident, based on multivariate logistic regression models shown in Table S5.

The plot (Figure 4) shows that in at-home cases of the asystole/PEA rhythm, the transition between age 74 and 75 was the breaking point between the odds favoring ROSC (age 74: odds ≈ 1.014) and death (age 75: odds ≈ 0.998). Conversely, the public place cases remained favorable to the development of ROSC, even at the age of 102 years old (odds ≈ 1.077). As the VF/pVT rhythm yields 3.746-fold higher odds compared to asystole/PEA, such a breaking point among the at-home cases could not be found for VF/pVT, as the odds would always favor ROSC. This observation accounts for the assumption that public location, in general, favors the development of ROSC, even at very advanced age, regardless of the initial rhythm. The ranges of odds of ROSC for different age groups, depending on initial rhythm and location, are shown in Figure 5.

## 4. Discussion

Our study aimed to investigate the impact of incident location (public vs. private places), initial rhythm (VF/pVT vs. asystole/PEA), and patient age on the likelihood of ROSC during OHCA. Patients who experienced CA in public locations had significantly higher odds of achieving ROSC compared to those whose incidents occurred at home (54.10% vs. 31.53%, *p* < 0.001).

Within the studied cohort, the interaction between incident location and initial rhythm was significant. Patients presenting with an initial shockable rhythm had higher odds of ROSC regardless of the location, with this effect being more pronounced in public places. Prior research suggests that individuals experiencing CA in public locations have a 4–5 times greater chance of survival than those experiencing it at home. Improved outcomes in public places may be attributed to quicker bystander response and the availability of AEDs [18,19]. While public locations benefit from faster access to AEDs and bystander intervention [20], home settings often face significant delays in the initiation of resuscitation [21,22]. This discrepancy is largely due to the fact that AEDs are rarely available in private residences, as individuals typically do not purchase them for home use, and there is often an absence of trained bystanders. Even when family members are present, the initiation of CPR may be delayed due to a lack of training or uncertainty about how to respond [23,24]. These delays can result in worse outcomes, particularly in cases where early defibrillation is critical. The association between initial VF/pVT rhythm and increased survival is well-established. For instance, Okubo et al., in a 26-year observation of OHCA, demonstrated that an initial shockable rhythm increases survival chances more than three-fold [25,26,27,28,29].

Specifically, patients under 60 years of age had better odds of ROSC when OHCA occurred at home, possibly due to the quicker intervention by family members. Conversely, in individuals over 60 years of age, the odds of ROSC were higher in public settings. Each additional year of age was significantly associated with a 1.62% decrease in ROSC odds for at-home incidents and a 0.40% decrease for incidents in public places, regardless of initial rhythm. This age–location interaction suggests that younger patients might benefit more from the familiarity and immediate availability of family members at home, who can initiate resuscitation efforts. Conversely, older patients might fare better in public places due to the quicker response times and potentially greater access to bystander CPR and emergency services in these settings. Additionally, older adults often live alone or away from immediate family, which can delay the initiation of resuscitation efforts when OHCA occurs at home [30,31]. This trend is consistent with findings from Holmstrom et al. and Chen et al., who reported that public locations can mitigate the negative impact of advanced age on ROSC outcomes. Furthermore, studies have shown that the availability of prompt medical intervention in public places, where emergency services are more readily accessible, significantly enhances survival rates among older adults experiencing CA. Similar findings were reported by Holmstrom et al. and Chen et al., suggesting that incident location can mitigate the negative impact of age on ROSC outcomes [7,10].

The interaction between incident location and sex also revealed that in public places, women had higher odds of ROSC compared to men. This discrepancy may be due to various social and biological factors. Studies regarding location and sex present mixed results. Yan et al., in their meta-analysis, observed that women more frequently experience OHCA at home, where bystanders are less likely to initiate CPR, adversely affecting resuscitation outcomes [6]. Conversely, in public locations, women had higher odds of ROSC, potentially due to physiological differences, better responses to emergency interventions, or differing causes of OHCA compared to men [32].

Rob et al. found that women admitted to the cardiology department after OHCA had different etiologies of cardiac arrest, received less aggressive treatment, and had worse survival rates and neurological outcomes compared to men [33]. Feng et al., in their meta-analysis, noted that women were older, less likely to experience OHCA in public locations, less likely to present with an initial shockable rhythm, and less frequently received bystander CPR compared to men [34]. In our study group, the initial rhythm combined with patient sex significantly influenced ROSC outcomes. Women presenting with VF/pVT rhythm had higher odds of ROSC in public places compared to men. Myat et al. suggest that emergency interventions for VF/pVT rhythm are more effective in women in public places, potentially due to better hemodynamic responses and lower vascular resistance in women [1,35,36].

This study has several limitations inherent to its retrospective observational design. First, the data were entered directly by EMS personnel into the electronic system immediately after completing medical procedures, without external verification or cross-checking. Therefore, the accuracy and completeness of the data rely on the documentation provided by EMS personnel during emergency situations, which may vary. While the data largely conform to the Utstein definitions for OHCA reporting [37], certain critical variables—such as the quality of bystander CPR and precise EMS response times—were not consistently available across all cases. These factors are crucial confounders that could have affected the study results, but their absence limited our ability to adjust for them in the analysis.

Second, the study spans a period of one and a half years, during which EMS protocols and public health interventions may have changed. These temporal changes, such as updated resuscitation guidelines or shifts in public health strategies, were not accounted for in the analysis. Notably, the study period overlapped with the COVID-19 pandemic, which may have significantly influenced OHCA outcomes [38,39]. Research has shown that the pandemic led to fewer instances of bystander CPR, longer EMS response times, and increased OHCA incidence rates, all of which negatively impacted survival outcomes [38,40]. The fear of contracting COVID-19 likely deterred bystanders from initiating CPR, and the additional precautions taken by EMS personnel, such as donning personal protective equipment (PPE), likely delayed response times [41]. These pandemic-related factors were not specifically measured in our dataset, limiting our ability to assess their direct impact on the study’s findings.

Despite these limitations, the study provides valuable insights into the factors influencing ROSC outcomes in OHCA patients in Poland. Future studies with more detailed datasets, including additional variables such as the quality of bystander CPR, EMS response times, and adjustments for changes in public health interventions, would be beneficial in further clarifying these relationships.

## 5. Conclusions

The interaction between the location of OHCA, the initial cardiac rhythm, and patient age significantly affects the likelihood of ROSC. Incidents in public places resulted in higher ROSC rates compared to those at home, especially in cases with an initial shockable rhythm (VF/pVT), where the effect was more pronounced in public locations. Furthermore, the impact of age on ROSC outcomes varied depending on location: patients under 60 years of age achieved better ROSC outcomes at home, while those over 60 showed higher ROSC rates in public places. These findings highlight the complex interaction between age, location, and rhythm in determining ROSC outcomes.

The findings of our study could support improvements in EMS protocols and public health strategies. The higher ROSC rates in public locations highlight the need to increase access to AEDs in residential areas, where these resources are less available. Additionally, public health campaigns should prioritize expanding CPR and AED training, especially for family members of high-risk individuals.

By strengthening existing community AED programs and ensuring that EMS protocols prioritize rapid AED deployment, we can potentially reduce the time to intervention during cardiac arrests at home. This, combined with ongoing education efforts, could help close the gap in ROSC outcomes between public and private locations.

## Figures and Tables

**Table 1:** Characteristics of the study population sample stratified based on the incidence location.

Parameter		Location: at Home	Location: a Public Place	Total	*p*
		(n = 27,287)	(n = 6349)	(n = 33,636)	
Age [years]	Mean (SD)	69.30 (14.97)	61.29 (14.90)	61.29 (14.90)	<0.001
	Median (quartiles)	70.00 (61.00–81.00)	63.00 (51.00–71.00)	63.00 (51.00–71.00)	
	Range	18.00–105.00	18.00–102.00	18.00–102.00	
Sex	Male	17,517 (64.20%)	4117 (64.84%)	21,634 (64.31%)	0.336
Initial rhythm	VF/pVT	1902 (6.97%)	957 (15.07%)	2859 (8.50%)	<0.001
Obesity	Yes	879 (3.22%)	186 (2.93%)	1065 (3.17%)	0.231
DM	Yes	4409 (16.16%)	937 (14.76%)	5346 (15.89%)	0.006
CS	Yes	1465 (5.37%)	348 (5.48%)	1813 (5.39%)	0.722
HT	Yes	6082 (22.29%)	1378 (21.70%)	7460 (22.18%)	0.310
HF	Yes	2280 (8.36%)	490 (7.72%)	2770 (8.24%)	0.095
ACS	Yes	1038 (3.80%)	304 (4.79%)	1342 (3.99%)	<0.001
ROSC	Yes	8604 (31.53%)	3435 (54.10%)	12,039 (35.79%)	<0.001

Abbreviations: n, number of patients; ROSC, return of spontaneous circulation, DM, diabetes mellitus; CS, cerebral stroke; HT, atrial hypertension; HF, heart failure; ACS, acute coronary syndrome; VF/pVT, ventricular fibrillation/pulseless ventricular tachycardia.

**Table 2:** Characteristics of the study population sample stratified based on both incidence location and initial rhythm.

Parameter		VF/pVT (n = 1902)	Asystole/PEA (n = 25,385)	*p*
Location: at home (n = 27,287)				
Age [years]	Mean (SD)	65.68 (13.63)	69.57 (15.03)190	<0.001
	Median (quartiles)	67.00 (58.00–74.00)	71.00 (62.00–81.00)	
	Range	19.00–99.00	18.00–105.00	
Sex	Male	1458 (76.66%)	16,059 (63.27%)	<0.001
Obesity	Yes	44 (2.31%)	835 (3.29%)	0.020
DM	Yes	217 (11.41%)	4192 (16.52%)	<0.001
CS	Yes	86 (4.52%)	1379 (5.43%)	0.089
HT	Yes	363 (19.09%)	5719 (22.53%)	<0.001
HF	Yes	122 (6.41%)	2158 (8.50%)	0.002
ACS	Yes	217 (11.41%)	821 (3.23%)	<0.001
ROSC	Yes	1168 (61.41%)	7436 (29.30%)	<0.001
Location: a public place (n = 6349)				
Age [years]	Mean (SD)	61.28 (12.87)	61.29 (15.23)	0.977
	Median (quartiles)	62.00 (54.00–70.00)	63.00 (50.00–71.00)	
	Range	18.00–97.00	18.00–102.00	
Sex	Male	745 (77.85%)	3372 (62.54%)	<0.001
Obesity	Yes	19 (1.99%)	167 (3.10%)	0.060
DM	Yes	97 (10.14%)	840 (15.58%)	<0.001
CS	Yes	41 (4.28%)	307 (5.69%)	0.078
HT	Yes	153 (15.99%)	1225 (22.72%)	<0.001
HF	Yes	55 (5.75%)	435 (8.07%)	0.013
ACS	Yes	126 (13.17%)	178 (3.30%)	<0.001
ROSC	Yes	759 (79.31%)	2676 (49.63%)	<0.001

Abbreviations: n, number of patients; ROSC, return of spontaneous circulation, DM, diabetes mellitus; CS, cerebral stroke; HT, atrial hypertension; HF, heart failure; ACS, acute coronary syndrome; VF/pVT, ventricular fibrillation/pulseless ventricular tachycardia.

**Table 3:** Odds ratios of ROSC in the context of incident location—based on multi-factor-adjusted logistic regression models.

Model	Feature	Category	Location: at Home		Location: a Public Place	
			OR	*p*	OR	*p*
Initial rhythm only	Initial rhythm	VF/pVT	3.840	<0.001	3.891	<0.001
Adjusted by initial rhythm, sex, and age	Initial rhythm	VF/pVT	3.715	<0.001	3.894	<0.001
	Sex	Male	0.979	0.449	0.961	0.463
	Age	-	0.984	<0.001	0.996	0.011
Adjusted by all featured factors	Initial rhythm	VF/pVT	3.739	<0.001	3.746	<0.001
	Sex	Male	0.980	0.462	0.950	0.344
	Age	-	0.984	<0.001	0.996	0.009
	Obesity	Yes	0.847	0.036	0.890	0.451
	DM	Yes	1.032	0.413	0.926	0.314
	CS	Yes	0.991	0.883	1.282	0.032
	HT	Yes	0.976	0.473	0.934	0.303
	HF	Yes	1.054	0.286	0.849	0.099
	ACS	Yes	0.930	0.304	1.449	0.006

More thorough information on these models is given in Supplementary Materials, Table S1. Abbreviations: ROSC, return of spontaneous circulation; DM, diabetes mellitus; CS, cerebral stroke; HT, atrial hypertension; HF, heart failure; ACS, acute coronary syndrome; VF/pVT, ventricular fibrillation/pulseless ventricular tachycardia.

**Table 4:** Odds ratios of ROSC in the context of incident location and initial rhythm—based on multi-factor-adjusted logistic regression models.

Location	Model	Feature	Category	Initial Rhythm:		Initial Rhythm:	
				Asystole/PEA		VF/pVT	
				OR	*p*	OR	*p*
Location: at home	Adjusted by sex and age Adjusted by all featured factors	Sex	Male	0.979	0.468	0.971	0.794
		Age	-	0.984	<0.001	0.980	<0.001
		Sex	Male	0.980	0.478	0.971	0.798
		Age	-	0.984	<0.001	0.980	<0.001
		Obesity	Yes	0.883	0.125	0.441	0.011
		DM	Yes	1.012	0.757	1.399	0.040
		CS	Yes	0.993	0.912	0.973	0.907
		HT	Yes	0.980	0.565	0.944	0.655
		HF	Yes	1.056	0.280	1.014	0.946
		ACS	Yes	0.925	0.333	0.956	0.766
Location: public place	Adjusted by sex and age Adjusted by all featured factors	Sex	Male	1.001	0.992	0.569	0.009
		Age	-	0.995	0.012	0.997	0.578
		Sex	Male	0.988	0.829	0.574	0.011
		Age	-	0.995	0.010	0.996	0.567
		Obesity	Yes	0.917	0.590	0.689	0.488
		DM	Yes	0.887	0.132	1.641	0.121
		CS	Yes	1.253	0.060	1.756	0.252
		HT	Yes	0.937	0.349	0.954	0.837
		HF	Yes	0.839	0.092	1.015	0.965
		ACS	Yes	1.430	0.022	1.513	0.119

More thorough information on these models is given in Supplementary Materials, Table S2. Abbreviations; ROSC, return of spontaneous circulation; DM, diabetes mellitus; CS, cerebral stroke; HT, atrial hypertension; HF, heart failure; ACS, acute coronary syndrome; VF/pVT, ventricular fibrillation/pulseless ventricular tachycardia.

## Data Availability

The data that support the findings of this study are available from the corresponding author upon reasonable request.

## Supplementary Materials

The following supporting information can be downloaded at: https://www.mdpi.com/article/10.3390/jcm13216426/s1, Table S1. More thorough report concerning models shown in Figure 3; Table S2. More thorough report concerning models shown in Figure 4; Table S3. Assessment of multicollinearity between the variables based on variance inflation factor (VIF); Table S4. Insights from a three-way logistic regression model which shows how sex, location, and initial rhythm, together, modulate the odds of ROSC; Table S5. Logistic regression model that aimed to show how location and age together modulate the odds of ROSC, and that the initial rhythm does not influence this interaction.
