# Supplementary material for: The Interaction Effect of Age, Initial Rhythm, and Location on Outcomes After Out-of-Hospital Cardiac Arrest: A Retrospective Cohort Study
Source: J Clin Med. 2024 Oct 26;13(21):6426. doi: 10.3390/jcm13216426 (PMC11547014; doi:10.3390/jcm13216426)
Supplement: Supplementary file 1 [file jcm-13-06426-s001.zip › jcm-3276886-supplementary.pdf]

Supplementary Materials

Table S1. More thorough report concerning models shown in Figure 3

	Model	Feature	Category	OR	OR -95% CI	OR 95% CI	p
Location: at home	Initial rhythm only (D = 32922, AIC = 32926, BIC = 32942)	Initial rhythm	VF/pVT	**3.840**	3.488	4.228	**<0.001**
	Adjusted by Initial rhythm, sex and age (D = 32575, AIC = 32583, BIC = 32616)	Initial rhythm	VF/pVT	**3.715**	3.370	4.096	**<0.001**
		Sex	Male	0.979	0.927	1.034	0.449
		Age	-	**0.984**	0.982	0.985	**<0.001**
	Adjusted by all featured factors (D = 32568, AIC = 32588, BIC = 32670)	Initial rhythm	VF/pVT	**3.739**	3.389	4.126	**<0.001**
		Sex	Male	0.980	0.927	1.035	0.462
		Age	-	**0.984**	0.982	0.985	**<0.001**
		Obesity	Yes	**0.847**	0.725	0.989	**0.036**
		DM	Yes	1.032	0.957	1.113	0.413
		CS	Yes	0.991	0.881	1.115	0.883
		HT	Yes	0.976	0.912	1.043	0.473
		HF	Yes	1.054	0.957	1.160	0.286
		ACS	Yes	0.930	0.809	1.068	0.304
Location: a public place	Initial rhythm only (D = 8388, AIC = 8392, BIC = 8406)	Initial rhythm	VF/pVT	**3.891**	3.298	4.590	**<0.001**
	Adjusted by Initial rhythm, sex and age (D = 8381, AIC = 8389, BIC = 8416)	Initial rhythm	VF/pVT	**3.894**	3.296	4.600	**<0.001**
		Sex	Male	0.961	0.864	1.069	0.463
		Age	-	**0.996**	0.992	0.999	**0.011**
	Adjusted by all featured factors (D = 8363, AIC = 8383, BIC = 8450)	Initial rhythm	VF/pVT	**3.746**	3.166	4.433	**<0.001**
		Sex	Male	0.950	0.853	1.057	0.344
		Age	-	**0.996**	0.992	0.999	**0.009**
		Obesity	Yes	0.890	0.657	1.206	0.451
		DM	Yes	0.926	0.797	1.076	0.314
		CS	Yes	**1.282**	1.022	1.607	**0.032**
		HT	Yes	0.934	0.820	1.064	0.303
		HF	Yes	0.849	0.699	1.031	0.099
		ACS	Yes	**1.449**	1.114	1.885	**0.006**

Abbreviations: AIC, Akaike Information Criterion; BIC, Bayesian Information Criterion; n, number of patients; ROSC, return of spontaneous circulation, D, deviance; DM, diabetes mellitus; CS, cerebral stroke; HT, atrial hypertension; HF, heart failure; ACS, acute coronary syndrome; VF/pVT — ventricular fibrillation/pulseless ventricular

Table S2. More thorough report concerning models shown in Figure 4

	Initial rhythm: Asystole/PEA							Initial rhythm: VF/pVT						
	Model	Feature	Category	OR	OR -95% CI	OR 95% CI	p	Model	Feature	Category	OR	OR -95% CI	OR 95% CI	p
Location: at home	Adjusted by sex and age (D = 30093, AIC = 30100, BIC = 30124)	Sex	Male	0.979	0.925	1.036	0.468	Adjusted by sex and age (D = 7413, AIC = 7419, BIC = 7439)	Sex	Male	0.971	0.778	1.212	0.794
		Age	-	**0.984**	0.982	0.986	**<0.001**		Age	-	**0.980**	0.973	0.987	**<0.001**
	Adjusted by all featured factors (D = 30089, AIC = 30107, BIC = 30180)	Sex	Male	0.980	0.925	1.037	0.478	Adjusted by all featured factors (D = 7397, AIC = 7415, BIC = 7474)	Sex	Male	0.971	0.778	1.213	0.798
		Age	-	**0.984**	0.982	0.986	**<0.001**		Age	-	**0.980**	0.973	0.987	**<0.001**
		Obesity	Yes	0.883	0.753	1.035	0.125		Obesity	Yes	0.441	0.234	0.832	**0.011**
		DM	Yes	1.012	0.936	1.095	0.757		DM	Yes	**1.399**	1.016	1.927	**0.040**
		CS	Yes	0.993	0.879	1.122	0.912		CS	Yes	0.973	0.618	1.533	0.907
		HT	Yes	0.980	0.914	1.050	0.565		HT	Yes	0.944	0.735	1.213	0.655
		HF	Yes	1.056	0.956	1.166	0.280		HF	Yes	1.014	0.683	1.504	0.946
		ACS	Yes	0.925	0.789	1.083	0.333		ACS	Yes	0.956	0.710	1.286	0.766
Location: at public place	Adjusted by sex and age (D = 2481, AIC = 2487, BIC = 2503)	Sex	Male	1.001	0.896	1.118	0.992	Adjusted by sex and age (D = 961, AIC = 967, BIC = 982)	Sex	Male	**0.569**	0.372	0.868	**0.009**
		Age	-	**0.995**	0.992	0.999	**0.012**		Age	-	0.997	0.984	1.009	0.578
	Adjusted by all featured factors (D = 2471, AIC = 2489, BIC = 2538)	Sex	Male	0.988	0.884	1.104	0.829	Adjusted by all featured factors (D = 953, AIC = 971, BIC = 1015)	Sex	Male	**0.574**	0.375	0.878	**0.011**
		Age	-	**0.995**	0.992	0.999	**0.010**		Age	-	0.996	0.984	1.009	0.567
		Obesity	Yes	0.917	0.668	1.258	0.590		Obesity	Yes	0.689	0.240	1.973	0.488
		DM	Yes	0.887	0.758	1.037	0.132		DM	Yes	1.641	0.877	3.069	0.121
		CS	Yes	1.253	0.991	1.586	0.060		CS	Yes	1.756	0.670	4.602	0.252
		HT	Yes	0.937	0.818	1.074	0.349		HT	Yes	0.954	0.609	1.494	0.837
		HF	Yes	0.839	0.684	1.029	0.092		HF	Yes	1.015	0.509	2.025	0.965
		ACS	Yes	**1.430**	1.052	1.944	**0.022**		ACS	Yes	1.513	0.899	2.547	0.119

Abbreviations: AIC, Akaike Information Criterion; BIC, Bayesian Information Criterion; n, number of patients; ROSC, return of spontaneous circulation; D, deviance; DM, diabetes mellitus; CS, cerebral stroke; HT, atrial hypertension; HF, heart failure; ACS, acute coronary syndrome; VF/pVT — ventricular fibrillation/pulseless ventricular tachycardia

Table S3. Assessment of multicollinearity between the variables based on Variance Inflation Factor (VIF)

Variable	VIF
Age	1.047
Location	1.055
Initial rhythm	1.040
Obesity	1.031
Diabetes	1.119
Stroke	1.017
Hypertension	1.127
Heart failure	1.039
Heart infarction	1.029
Sex	1.016

Table S4. Insights from a three-way logistic regression model which shows how sex, location and initial rhythm, together, modulate the odds of ROSC

A. Full factorial model: three-way influence of sex, Initial rhythm and location on odds of ROSC											
Effect/interaction	Analyzed cat.	Reference cat.	β	β SE	Wald stat.	β -95% CI	β 95% CI	OR	OR -95% CI	OR 95% CI	p
Intercept	-	-	0.468	0.098	23.010	0.277	0.659	**1.596**	1.319	1.933	**<0.001**
Location	A public place	At home	1.335	0.220	36.870	0.904	1.766	**3.800**	2.470	5.847	**<0.001**
Initial rhythm	Asystole/PEA	VF/pVT	-1.331	0.100	176.833	-1.528	-1.135	**0.264**	0.217	0.321	**<0.001**
Sex	Male	Female	-0.004	0.111	0.001	-0.223	0.214	0.996	0.800	1.239	0.969
Location*Initial rhythm	-	-	-0.489	0.225	4.707	-0.931	-0.047	**0.613**	0.394	0.954	**0.030**
Location*Sex	-	-	-0.565	0.243	5.412	-1.040	-0.089	**0.569**	0.353	0.915	**0.020**
Initial rhythm*Sex	-	-	-0.023	0.115	0.041	-0.249	0.202	0.977	0.780	1.224	0.840
Location*Initial rhythm*Sex	-	-	0.597	0.251	5.666	0.105	1.089	**1.817**	1.111	2.970	**0.017**

B. Three-way interactions based on the above model, part 1: odds ratios												
Conditions	Analyzed var.	Analyzed cat.	Reference cat.	β	β SE	Wald stat.	β -95% CI	β 95% CI	OR	OR -95% CI	OR 95% CI	p
Initial rhythm: Asystole/PEA; Sex: Female	Location	A public place	At home	0.846	0.050	286.797	0.748	0.944	**2.330**	2.113	2.570	**<0.001**
Initial rhythm: VF/pVT; Sex: Female	Location	A public place	At home	1.335	0.220	36.870	0.904	1.766	**3.800**	2.470	5.847	**<0.001**
Initial rhythm: Asystole/PEA; Sex: Male	Location	A public place	At home	0.878	0.039	518.165	0.803	0.954	**2.406**	2.231	2.595	**<0.001**
Initial rhythm: VF/pVT; Sex: Male	Location	A public place	At home	0.770	0.103	56.094	0.569	0.972	**2.160**	1.766	2.643	**<0.001**
Location: a public place; Sex: Female	Initial rhythm	Asystole/PEA	VF/pVT	-1.821	0.202	81.226	-2.217	-1.425	**0.162**	0.109	0.241	**<0.001**

Location: at home; Sex: Female	Initial rhythm	Asystole/PEA	VF/pVT	-1.331	0.100	176.833	-1.528	-1.135	**0.264**	0.217	0.321	**<0.001**
Location: a public place; Sex: Male	Initial rhythm	Asystole/PEA	VF/pVT	-1.247	0.094	175.256	-1.432	-1.062	**0.287**	0.239	0.346	**<0.001**
Location: at home; Sex: Male	Initial rhythm	Asystole/PEA	VF/pVT	-1.355	0.057	574.353	-1.466	-1.244	**0.258**	0.231	0.288	**<0.001**
Location: a public place; Initial rhythm: Asystole/PEA	Sex	Male	Female	0.005	0.056	0.007	-0.106	0.115	1.005	0.900	1.122	0.932
Location: at home; Initial rhythm: Asystole/PEA	Sex	Male	Female	-0.028	0.029	0.930	-0.084	0.028	0.973	0.920	1.029	0.335
Location: a public place; Initial rhythm: VF/pVT	Sex	Male	Female	-0.569	0.216	6.959	-0.992	-0.146	**0.566**	0.371	0.864	**0.008**
Location: at home; Initial rhythm: VF/pVT	Sex	Male	Female	-0.004	0.111	0.001	-0.223	0.214	0.996	0.800	1.239	0.969
C. Three-way interactions based on the above model, part 2: (ratios of) odds ratios												
Conditions, covariate	Interaction	Categories: variable 1	Categories: variable 2	β	β SE	Wald stat.	β -95% CI	β 95% CI	OR	OR -95% CI	OR 95% CI	p
Initial rhythm: Asystole/PEA	Location*Sex	A public place vs. At home	Male vs. Female	0.032	0.063	0.262	-0.091	0.156	1.033	0.913	1.169	0.609
Initial rhythm: VF/pVT	Location*Sex	A public place vs. At home	Male vs. Female	-0.565	0.243	5.412	-1.040	-0.089	**0.569**	0.353	0.915	**0.020**
Location: A public place	Initial rhythm*Sex	Asystole/PEA vs. VF/pVT	Male vs. Female	0.574	0.223	6.625	0.137	1.011	**1.775**	1.147	2.747	**0.010**
Location: At home	Initial rhythm*Sex	Asystole/PEA vs. VF/pVT	Male vs. Female	-0.023	0.115	0.041	-0.249	0.202	0.977	0.780	1.224	0.840
Sex: Female	Initial rhythm*Location	Asystole/PEA vs. VF/pVT	A public place vs. At home	-0.489	0.225	4.707	-0.931	-0.047	**0.613**	0.394	0.954	**0.030**
Sex: Male	Initial rhythm*Location	Asystole/PEA vs. VF/pVT	A public place vs. At home	0.108	0.110	0.963	-0.107	0.323	1.114	0.898	1.381	0.326

'X*Y' terms denote interactions between variables (effects). P-values lower than 0.05 are marked **in bold**. Abbreviations: CI, confidence interval; OR, odds ratio; SE, standard error.
Insights from parts B and C of this table is visualized in Figure 1. Estimated odds of ROSC (intercept) upon variable conditions are featured in Figure 2.

Table S5. Logistic regression model which aimed to show how location and age together modulate the odds of ROSC, and that the initial rhythm does not influence this interaction

A. Full factorial model: three-way influence of age, location and initial rhythm on ROSC								
Effect/interaction	Analyzed cat.	Reference cat.	β	β SE	Wald stat.	β -95% CI	β 95% CI	p
Intercept	-	-	**0.245**	0.062	15.764	0.124	0.367	**<0.001**
Location	A public place	At home	0.011	0.126	0.008	-0.235	0.257	0.929
Age	-	-	**-0.016**	0.001	344.867	-0.018	-0.015	**<0.001**
Location*Age	-	-	**0.012**	0.002	38.171	0.008	0.016	**<0.001**
Initial rhythm	VF/pVT	Asystole/PEA	**1.310**	0.050	696.199	1.212	1.407	**<0.001**
Location*Initial rhythm*Age	-	-	0.044	0.098	0.199	-0.149	0.236	0.655

B. Analysis of the Location*Age interaction, adjusted for initial rhythm and its interaction with age						
Location: at home						
Effect/interaction	Analyzed cat.	Reference cat.	OR	OR -95% CI	OR 95% CI	p
Baseline odds of ROSC (age: 60)	-	-	**1.277**	1.131	1.441	**<0.001**
Fold change in odds upon 1-year increase in age	-	-	**0.984**	0.982	0.985	**<0.001**
Fold change in odds upon 1-year decrease in age	-	-	**1.016**	1.018	1.015	**<0.001**
Difference between initial rhythms at age 60	VF/pVT	Asystole/PEA	**3.746**	3.445	4.074	**<0.001**
Change in OR between initial rhythms upon 1-year increase in age	VF/pVT	Asystole/PEA	0.997	0.991	1.003	0.372
Location: a public place						
Effect/interaction	Analyzed cat.	Reference cat.	OR	OR -95% CI	OR 95% CI	p
Baseline odds of ROSC (age: 60)	-	-	**1.297**	1.047	1.606	**0.017**
Fold change in odds upon 1-year increase in age	-	-	**0.996**	0.992	0.999	**<0.001**
Fold change in odds upon 1-year decrease in age	-	-	**1.004**	1.008	1.001	**<0.001**
Difference between initial rhythms at age 60	VF/pVT	Asystole/PEA	**3.746**	3.445	4.074	**<0.001**
Change in OR between initial rhythms upon 1-year increase in age	VF/pVT	Asystole/PEA	0.997	0.991	1.003	0.372
